# A modified Delphi process to establish research priorities in hernia surgery

**DOI:** 10.1007/s10029-021-02519-0

**Published:** 2021-10-31

**Authors:** D. S. G. Scrimgeour, M. Allan, S. R. Knight, B. East, S. Blackwell, N. Dames, L. Laidlaw, D. Light, L. Horgan, N. J. Smart, A. de Beaux, M. S. J. Wilson, on behalf of the Scottish Surgical Research Group and the British Hernia Society

**Affiliations:** 1 Department of Colorectal Surgery, Aberdeen Royal Infirmary, Aberdeen, AB25 2ZN Scotland; 2 Institute of Genetics and Cancer, University of Edinburgh, Edinburgh, EH4 2XU Scotland; 3 Centre for Medical Informatics, Usher Institute, University of Edinburgh, Edinburgh, EH16 4UX Scotland; 4 3rd Department of Surgery, Motol University Hospital, V Uvalu 84, 150 06 Prague, Czech Republic; 5 Patient Representative, Liverpool, UK; 6 Patient Representative, Glasgow, UK; 7 Patient Representative, Edinburgh, UK; 8 Department of UGI Surgery, Northumbria Healthcare NHSFT, Rake Ln, Tyne and Wear, North Shields, NE29 8NH UK; 9 Department of Gastrointestinal Surgery, Royal Devon and Exeter NHS Trust, Barrack Road, Exeter, EX25DW Devon UK; 10 Department of General Surgery, Royal Infirmary of Edinburgh, Edinburgh, EH16 4SA Scotland; 11 Department of General Surgery, Forth Valley Royal Hospital, Larbert, FK5 4WR UK

**Keywords:** Delphi, Hernia, Surgery, Registry

## Abstract

**Background:**

Abdominal wall hernia repair is one of the most commonly performed surgical procedures worldwide, yet despite this, there remains a lack of high-quality evidence to support best management. The aim of the study was to use a modified Delphi process to determine future research priorities in this field.

**Methods:**

Stakeholders were invited by email, using British Hernia Society membership details or Twitter, to submit individual research questions via an online survey. In addition, questions obtained from a patient focus group (PFG) were collated to form Phase I. Two rounds of prioritization by stakeholders (phases II and III) were then completed to determine a final list of research questions. All questions were analyzed on an anonymized basis.

**Results:**

A total of 266 questions, 19 from the PFG, were submitted by 113 stakeholders in Phase I. Of these, 64 questions were taken forward for prioritization in Phase II, which was completed by 107 stakeholders. Following Phase II analysis, 97 stakeholders prioritized 36 questions in Phase III. This resulted in a final list of 14 research questions, 3 of which were from the PFG. Stakeholders included patients and healthcare professionals (consultant surgeons, trainee surgeons and other multidisciplinary members) from over 27 countries during the 3 phases.

**Conclusion:**

The study has identified 14 key research priorities pertaining to abdominal wall hernia surgery. Uniquely, these priorities have been determined from participation by both healthcare professionals and patients. These priorities should now be addressed by well-designed, high-quality international collaborative research.

**Supplementary Information:**

The online version contains supplementary material available at 10.1007/s10029-021-02519-0.

## Introduction

Abdominal wall hernias are common and hernia repair is one of the most commonly performed operations in the world, generating huge health care costs. Indeed, the incidence of inguinal hernias, primary ventral hernias and incisional hernias are rising due to an aging population and the world obesity crisis [1, 2]. However, despite this phenomenon there is a persistent lack of high-quality evidence regarding the management of abdominal wall hernias.

Delphi methodology is a well-established process used to collate judgements and opinions on a specific topic [3]. The process is dependent on obtaining consensus amongst a group of experts within the area of research interest. This methodology has been successfully utilized to identify research priorities for many surgical specialties including: colorectal surgery [3], plastic surgery [4], upper gastrointestinal surgery [5–7], and hepato-pancreato-biliary surgery [8]. Identifying the direction that future research should take can help to guide funding bodies and channel resources.

Modified Delphi techniques have also been used within the field of hernia surgery. Recent consensus has helped to provide definitions for loss of domain [9] and for the classification of abdominal wall planes to describe mesh insertion [10]. Convalescence recommendations following inguinal hernia repair [11] and management of chronic post-operative inguinal pain [12] have also been established using a Delphi process, but to the best of our knowledge, no previous attempt has been made to determine what the future research priorities in hernia surgery should be.

We, therefore, used a modified Delphi process with the aim to identify and prioritize future research questions in current hernia practice that are deemed to be of greatest clinical importance to patients, surgeons and allied health professionals. The study was undertaken as a collaboration between the Scottish Surgical Research Group (SSRG) and the British Hernia Society (BHS).

## Methods

**Fig. 1:**
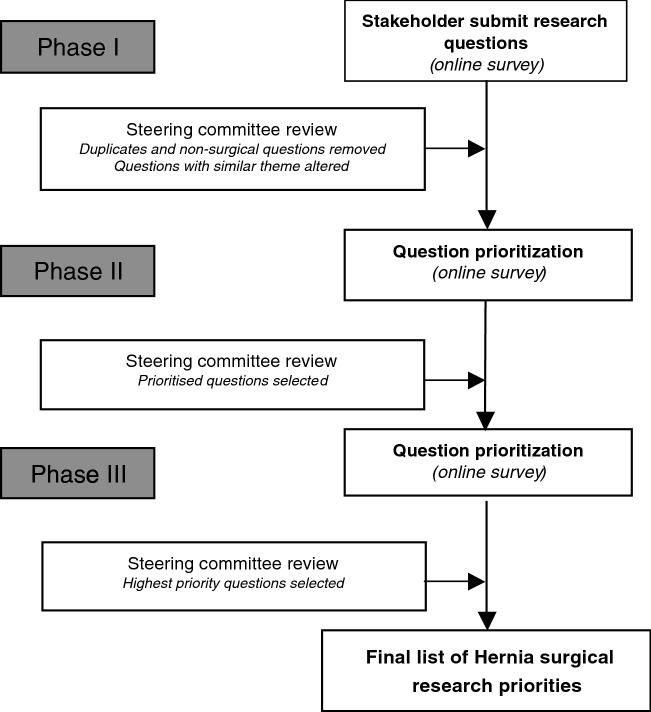
Three phase Delphi methodology

This study adopted a three-phase modified Delphi technique (Fig. 1) to establish research priorities within the field of hernia surgery, with the exclusion of hiatal hernia surgery. Two distinct phases of prioritization were conducted by expert multidisciplinary stakeholders using previously described and well-established methodology [6–8]. Stakeholders were initially invited to submit questions and then to prioritize their responses based upon their perceived clinical relevance and importance. Incomplete submissions where all questions were not ranked were excluded from the analysis of the prioritization phases (II and III).

### Phase I

Members of the BHS were invited by email to submit research focused hernia surgery questions via an online survey (www.surveymonkey.co.uk^®^). BHS members include surgeons, nurses, patients and other allied health professionals. A dedicated Twitter^®^ account (@hernia_delphi) was also used to increase the awareness of the study and encourage wider stakeholder participation. Stakeholders were invited to submit as many questions as they wished. The survey remained open for 6 weeks between October and December 2019. Five dedicated hernia Delphi tweets were made from the official hernia Delphi account and two email reminders to the BHS membership were sent during this period.

### Patient Focus Group (PFG)

Ten patients participated in a unique hernia Focus Group held at the Royal College of Surgeons of Edinburgh in February 2020. This session was facilitated by two (LL and ND) of the steering committee patient and public involvement (PPIs) members who helped guide the group discussion. Patients were invited via a Twitter advertisement and through direct invitation via outpatient clinic from members of the steering committee. Recurring themes that were deemed to be important to the majority of PFG participants were identified and collated during the course of the day.

The completion of the PFG marked the end of Phase I. Submitted questions were analyzed by a subcommittee. Duplicate questions were removed and recurring theme questions were altered by consensus agreement of the steering committee to ensure that the meaning of the questions were not distorted. The themes identified from the PFG were subsequently converted into research questions and included in the next phase of the Delphi process.

### Phase II

During Phase II stakeholders were invited, via email and Twitter, to rank the Phase I questions using a Likert scale from 1 (lowest research priority) to 5 (highest research priority). Five reminders were sent via Twitter and by two emails to the BHS membership over a 6 week period between August and October 2020. The steering committee reviewed the results whilst blinded to the questions and selected a mean ‘cut-off’ score of ≥ 3.60 for inclusion in the final phase of the study.

### Phase III

During the final Phase of the study, stakeholders were again invited, via email and Twitter, to rank the questions brought forward from Phase II using the same Likert scale as previously described. The survey remained open for 6 weeks between October and December 2020. Five reminder Tweets were made and two email reminders sent to BHS members. An invitation to complete the survey was also advertised during the BHS 2020 virtual conference. A final mean ‘cut-off’ score of ≥ 4.0 was agreed by the steering committee as described in Phase II.

### Steering committee

The committee consisted of three SSRG general surgery specialty registrars (DS, SRK and MA), an international hernia research fellow (BE), three PPIs (ND, LL and SB) and four general surgeons who perform high-volume hernia surgery (MW, DL, NS and AB) as BHS members. All decisions in relation to methodology and analysis were agreed on a consensus basis.

## Results

**Fig. 2:**
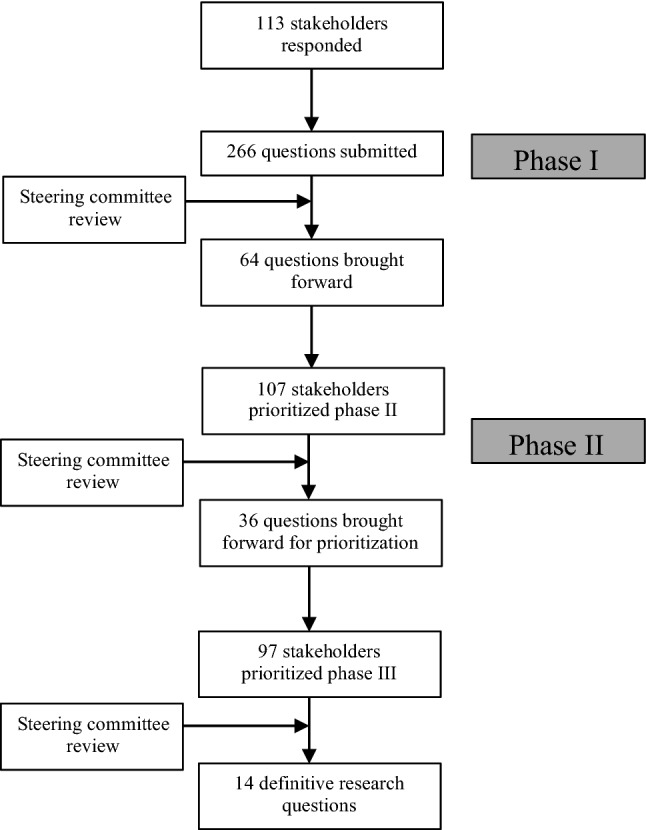
Overview of responses for modified Delphi survey

**Fig. 3:**
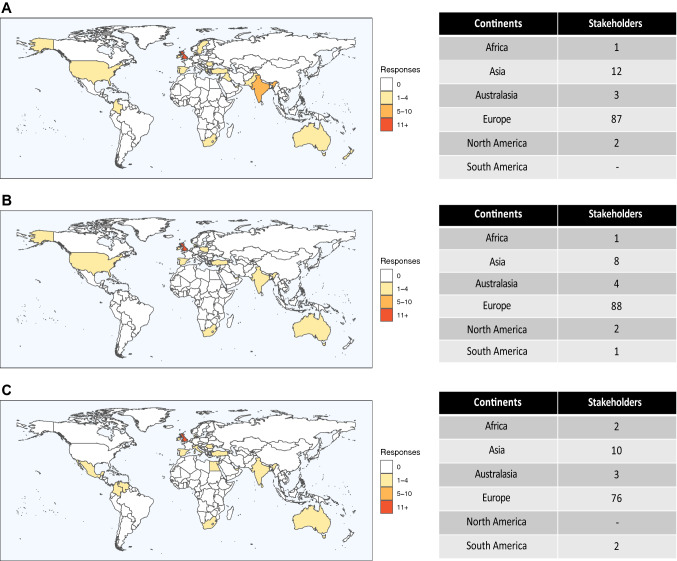
Geographical responses of stakeholders from each phase. **A**—Phase I, **B**—Phase II, **C**—Phase III

A total of 247 individual research questions were submitted online by 103 stakeholders during Phase I. With the addition of 19 questions proposed by the PFG, the final Phase I cohort totaled 266 research questions from 113 stakeholders (Fig. 2). Stakeholders were from 19 countries (Fig. 3A) and included patients (*n* = 19) and healthcare professionals (*n* = 94); professor or consultant surgeon (71.3%), surgical trainee/fellow/specialty doctor (25.5%), other (3.2%). Following analysis and categorization by the steering committee, 64 questions were moved forward for prioritization in Phase II.

One hundred and seven stakeholders from 14 countries (Fig. 3B) prioritized the questions in Phase II. Stakeholders comprised patients (*n* = 12) and healthcare professionals (*n* = 95), professor or consultant surgeon (77.9%), surgical trainee/fellow/specialty doctor (15.8%), other (6.3%). A total of 36 questions were included in Phase III.

Phase III comprised 97 stakeholders from 16 countries (Fig. 3C) and included patients (*n* = 11) and healthcare professionals (*n* = 86), professor or consultant (74.4%), surgical trainee/fellow/specialty doctor (23.3%), other (2.3%) . Following this final prioritization, 14 questions met the criteria to be defined as high research priority (Table 1). Three questions submitted by the patient focus group were included in the final list of 14 high-research priority questions. The remaining 16 questions from the patient focus group, which did not make the cut-off, are detailed in Table 2. All questions which failed to make the final list of research priorities from Phase III are shown in Appendix 1.

**Table 1:** Final list of proritized research questions

Final list of prioritized questions
What data should be collected in a UK hernia registry?
What is the true incidence of complications following hernia surgery and is there a difference in complications between mesh and non-mesh groups? *Definition of incidence: the number of new cases (e.g. complications) over a set period of time*
What are the most important patient outcomes following hernia surgery?
**What are the most effective strategies for preventing and managing chronic pain after hernia surgery?
**What is the true incidence of chronic groin pain after hernia surgery (open and laparoscopic)? *Definition of incidence: the number of new cases (e.g. chronic groin pain) over a set period of time*
What are the most important outcome measures for incisional hernia repairs? *Definition of incisional hernia: a hernia that occurs at the site of a previous surgical incision or cut following surgery*
**Should the operating surgeon be informed of their recurrence or incisional hernia rate? *Definition of recurrence: the reappearance of a hernia after it has previously been repaired* *Definition of incisional hernia: a hernia that occurs at the site of a previous surgical incision or cut following surgery*
Does the type and characteristics of the mesh reduce the incidence of complications following hernia surgery? *Definition of incidence: the number of new cases (e.g. complications) over a set period of time*
Does incisional hernia repair improve patient quality of life?
Do functional outcomes improve following incisional hernia repair and abdominal wall reconstructive surgery?
What are the optimal outcome measures following hernia surgery?
What are the long-term adverse consequences of IPOM? *Definition of IPOM: Intra Peritoneal Onlay Mesh. The mesh is placed from within the abdominal cavity and covers the opening of the hernia*
Why do some patients develop chronic pain after inguinal hernia surgery?
What are the most appropriate tools for collecting PROMS before and after hernia surgery? *Definition of PROMS: Patient-Reported Outcome Measures, used to assess the quality of care delivered to a patient, from the patients' perspective*

^**^Those submitted by the patient Focus Group

**Table 2:** PFG questions which did not make the final prioritized list

Remaining Patient Focus Group questions
Does the quality of the pre-op information affect the patient’s perceptions of QoLL ?
Does educating hernia patients improve their outcomes?
Does peer support before surgery improve patient outcomes?
Is there a role for a hernia database/patient-led database?
Should hernias be treated as a specialist subject and looked after by specialist surgeons in a hernia service?
Recurrent hernias - should they be referred back to the original surgeon?
Does an opt in group/clinic information session improve PROMS?
Would a regional post-hernia pain pathway improve long-term patient outcomes?
Should we make a validated pain tool for specific hernia surgery?
Are there standardized protocols within regions/hospitals for hernia service?
Should body image be a valid indication when considering possibility of hernia surgery?
Does surgery for cosmetic only purposes improve patient QoL?
Would videoing of the consent process improve patient outcomes?
Does patient interaction/option to ask questions whilst on waiting list improve outcomes - same question for the post-operative setting?
Would a hernia nurse improve outcomes of patient QoL - particularly in post-op period?
Would the role of a structured checklist for hernia surgery improve QoL?

## Discussion

This modified Delphi process has produced a list of 14 high priority research questions pertaining to the field of hernia surgery. Questions were submitted during the three phases by stakeholders from all six continents and thus reflects the requirements in surgical research worldwide, including the differing views of developed and developing countries.

To increase participant numbers, each phase was considered as a separate entity and stakeholders were able to enrol in the study during any of the three, thus explaining the variation in participant numbers during the study. The authors attempted to avoid research fatigue by leaving a significant time gap between the phases, however, there was a small number of participants (*n* = 6) who did not continue from Phase II to III.

Unique to this Delphi process was a PFG who identified 19 research questions that were deemed to be of greatest importance to patients. Three of these questions were prioritized in the third phase to be included in the final list (Table 1). Whilst there was some overlap between surgeon and patient questions, in particular relating to the incidence and management of chronic groin pain, there were also some major disparities. A large proportion of questions submitted by the PFG (*n* = 5) focused on pre-operative information, whether that included peer support groups, pre-operative education, videoing of consent or patient interaction whilst on the waiting list. Interestingly, this is a topic that was not prioritized to the final list of questions, but is perhaps something that as a group of medical professionals we should be spending more time investigating in order to optimize the patient experience.

In addition to pre-op information, the question of hernia surgery for cosmetic indications was submitted by the PFG but it did not make the final list. This is undoubtedly a controversial topic; the risk versus benefits of hernia surgery for cosmesis would perhaps preclude some medical professionals from offering surgery, however, it is clearly something that is important to the patient community.

Unsurprisingly, many of the research topics focused on pain, mesh and what the optimal outcome measures following hernia surgery should be. Additional questions were related to the United Kingdom (UK) hernia registry and whether surgeons should be informed of their recurrence and/or incisional hernia rates.

The question “Should the operating surgeon be informed of their recurrence or incisional hernia rate?” is an interesting but potentially contentious one that may generate more questions than answers. Although most surgeons and patients will likely want to know, the practicality of collecting and using this information to change practice may be challenging. Nonetheless, this information may encourage some surgeons to pay more attention during abdominal wall closure and highlight surgeons/units with outlying recurrent incisional hernia rates, which is associated with non-specialists performing hernia surgery [13]. However, the accurate detection of an incisional and recurrent hernia will require cross sectional imaging, as demonstrated by the HART trial which found computed tomography scanning to be superior to clinical examination. This may have a significant impact on health care resources [14].

The tension-free vaginal tape (TVT) controversy has had a significant impact on patients’ trust in their surgeon who wishes to perform a hernia repair with mesh [15]. These concerns are understandable given the recent media frenzy, but the evidence of lower recurrence rates for the use of mesh over non-mesh is compelling [16, 17]. However, to date, there are over 300 meshes available, each with unique characteristics that may help to guide which mesh should be used in which clinical scenario, for example during emergency surgery and in contaminated surgical fields [18]. It is, therefore, not surprising that mesh related research questions made the final prioritized list (Table 1).

At the time of the final prioritization process, there was a paucity of high-quality evidence on the long-term complications associated with IPOM, hence the inclusion of “What are the long-term adverse consequences of IPOM?” in the final list. However, Henriksen et al. have recently published a nationwide database study on the short- and long-term outcomes of IPOM, concluding that IPOM should still be considered for fascial defects between 2 and 6 cm [19]. The authors conducted this study on behalf of the Danish Hernia Database, a national clinical quality database established in 2007 which registers hernia operations carried out in Denmark. This registry has facilitated research in hernia surgery that has influenced clinical practice [20–22]. Several other European hernia registries now exist, with the United States of America becoming the most recent country to develop its hernia registry [23]. The UK’s hernia registry is in fruition with plans to pilot data entry later this year [24]. To this end, the top priority research question as identified from this Delphi process “What data should be collected in a UK hernia registry?” will hopefully be addressed.

The primary outcome measure for hernia surgery has traditionally focused on recurrence, but in recent years, there has been a paradigm shift towards a greater focus on patient satisfaction and PROMs following hernia surgery. This is reflected in the number of hernia-specific PROM questions that have featured in the final list of research questions selected during this Delphi process (Table 1).

Despite this shift towards patient-centered primary outcome measures in recent years, there remains significant inconsistencies in PROM reporting. This has been highlighted by two well conducted systematic reviews that were designed to examine outcome reporting in both inguinal and ventral hernia surgery [25, 26]. Both systematic reviews strongly support the notion that outcome reporting should consist of several key outcomes which are deemed important to both clinicians and patients alike.

The Core Outcome Measures in Effectiveness Trials (COMET) initiative has been designed to identify which PROMs should be included for both inguinal and incisional hernia repair [27]. This four-phase mixed-methods approach will help to establish Core Outcome Sets (COS) that will lead to consistent outcome reporting between studies, facilitating future high-quality hernia research.

Once these outcomes have been developed the next challenge will be to establish how to accurately collect and record them. Appropriate tools for collecting these outcomes will need to be developed to enable seamless integration into current and planned hernia registries. For this reason, the question “What are the most appropriate tools for collecting PROMS before and after hernia surgery?” was prioritized.

The development of a COS in incisional hernia is still in its infancy with the systematic review from the first phase of the process just recently published. Harji and colleagues identified a total of 2215 outcomes reported in incisional hernia surgery, which were mapped to eight broad-based domains [28]. It is unclear at such an early stage whether abdominal wall functional outcomes will feature in the final COS, but this outcome did not appear in their final list of outcomes extracted. Interestingly, both retro- and prospective studies have reported improved functional outcomes following incisional hernia repair which appears to correlate with an improved overall Quality of Life (QoL) [29, 30]. Certainly, recent studies have suggested a satisfaction rate of 50–86% at 5–10 year follow-up with respect to QoL [31]. However, although most of these single-center studies have been well conducted, they have involved small sample sizes from expert abdominal wall institutions which brings into question the generalisability of their results. “Do functional outcomes improve following incisional hernia repair and abdominal wall reconstructive surgery?” was, therefore, still deemed, by our Delphi participants to be an appropriate and important research question. To expand upon this question, we could investigate specifically which patients have the greatest improvement in QoL after incisional hernia repair.

In January 2018, the European Hernia Society (EHS) published comprehensive international guidelines for groin hernia management [32]. These guidelines included several detailed statements and recommendations on how to reduce chronic post-operative inguinal pain (CPIP, defined as pain lasting more than three months). Many of these recommendations are related to meticulous surgical technique combined with nerve anatomy awareness and recognition during surgery. A multidisciplinary approach to the management of CPIP has also been recommended but there is a paucity of evidence available on which treatment strategies are most effective. In addition, although several risk factors for CPIP have been identified: female gender; high pre-operative pain; young age; early high post-operative pain; recurrent hernia and open repair, the reasons behind the question “Why do some patients develop chronic pain after inguinal surgery” remains unknown. However, what is clear is that there is currently no evidence to suggest that mesh insertion is the cause [33]. On the contrary, a recent Cochrane review reported a higher rate of post-operative pain with suture repair [34].

The prevalence of CPIP has been quoted to be between 10 and 12% based on several studies dating back to the beginning of the twenty-first century, decreasing over time, with debilitating chronic pain affecting daily activities ranging from 0.5 to 0.6% [35, 36]. However, the recent publication of EHS groin hernia management guidelines should help to remind surgeons of some simple, yet effective intraoperative strategies to reduce post-operative pain. These include: minimizing surgical trauma to the spermatic cord; avoiding mesh fixation to the pubic tubercle; selected sac invagination and the pragmatic resection of the ilioinguinal and/or iliohypogastric nerve(s) if iatrogenic injury is likely. It may then be necessary to revisit the question prioritized during this Delphi study “What is the true incidence of chronic groin pain after hernia surgery?”.

### Limitations

Whilst this is the first hernia study to seek the views of patient representatives, in collaboration with healthcare providers, the proportion of stakeholders which were patients was still significantly lower than that of surgeons. We believe in order to truly define the key hernia research priorities, it is important to have equal weighting of both service users and surgeon’s views. Is important to acknowledge the selection bias that may have been introduced  during the PFG session as the majority of patients who attended had already undergone their hernia surgery and thus many of the questions initially put forward were related to post-surgery issues, specifically chronic pain. Whilst these are key areas to be researched, less weighting was placed on pre-operative care which is also important and may indeed impact on the long-term outcomes. In addition, despite stakeholders being from over 27 countries during the three phases, they were predominantly from more developed countries. Research priorities may be substantially different in the more developing countries and this was perhaps not relayed entirely in this study.

The authors acknowledge that some questions in the final list of research priorities are already being addressed, in particular those relating to hernia registries and PROMS. The initial phase of this study and PFG was undertaken prior to the COVID-19 pandemic, with subsequent phases unfortunately being delayed by almost a year to prioritize the clinical need of the pandemic over research. It is inevitable, therefore, due to the long time period the study ran over, that some of these questions will already have more evidence around them by the time of this study being published.

## Conclusions

In conclusion, we have undertaken a modified Delphi process to determine a list of abdominal wall hernia research priorities. The study involved a large body of multidisciplinary healthcare professionals across multiple centers worldwide. Unique to this hernia study is the direct involvement of patients during all three phases and the PFG. These priorities should now be addressed by well-designed, high-quality international collaborative research.

## Supplementary Information

Below is the link to the electronic supplementary material.

Supplementary file1 (DOCX 116 kb)

## Data Availability

Available on request.
