# Supplementary material for: A modified Delphi process to establish research priorities in hernia surgery
Source: Hernia. 2021 Oct 31;26(3):751–9. doi: 10.1007/s10029-021-02519-0 (PMC8557712; doi:10.1007/s10029-021-02519-0)
Supplement: Supplementary file 1 — Supplementary file1 (DOCX 116 kb) [file 10029_2021_2519_MOESM1_ESM.docx]

Appendix 1 – Questions from Phase III not prioritised to final list.

1. How can we educate/prepare and manage patient expectations for hernia surgery?
2. What is the immunological response to mesh?
3. What is the optimal post-operative advice for patients who have undergone ventral hernia repairs in terms of function/heavy lifting/pain etc?

Definition of ventral hernia = a hernia that occurs at a weak point in the muscles of the abdominal wall (e.g. epigastric / umbilical / paraumbilical hernias)

1. Would a regional post-hernia pain pathway improve long-term patient outcomes?
2. Should a validated pain tool be designed specifically for hernia surgery?
3. What are the long term outcomes of prophylactic mesh placement during stoma formation?

Define prophylactic mesh = the planned placement of mesh with the intention of preventing or reducing the risk of hernia formation in the future.

Define stoma = in this context means an artificial opening in the bowel which is brought out through the abdominal wall to allow the passage of urine or faeces through it.

1. Does the type and the characteristics of mesh reduce the incidence of post operative pain following hernia surgery?

Definition of incidence = the number of new cases (e.g. post operative pain) over a set period of time.

1. What is the natural history of an asymptomatic incisional hernia? (frequency of growth, symptoms, emergency complications
2. What is the optimal referral pathway and management for patients with chronic groin pain following groin hernia repair?
3. What are the indications for mesh removal in patients with chronic pain following mesh hernia repair?
4. Should body image be a valid indication to consider a patient for hernia surgery?
5. Does surgery for only cosmetic reasons improve patient quality of life?
6. Would the addition of a specialist hernia nurse improve the patient experience of the hernia surgery journey?
7. Should the original surgeon perform the surgical repair of a recurrent hernia or should they be referred to a hernia specialist team?
8. How should outcomes following hernia surgery be reported in the scientific literature?
9. What are the most effective ways to reduce post-operative seromas after hernia surgery?

Definition of seroma = a collection of fluid that builds up and can be felt under the surface of your skin after a surgical procedure (e.g. hernia repair)

1. What are the long term adverse consequences of IPOM?

Definition of IPOM = IntraPeritoneal Onlay Mesh. The mesh is placed from within the abdominal cavity and covers the opening of the hernia.

1. Does the quality of the pre-operative information affect the patient’s perceptions of quality of life following hernia surgery?
2. Does educating hernia patients in the pre-operative period improve their clinical outcomes?
3. Recurrent hernias – should they be referred to the original operating surgeon?

Definition of recurrence = the reappearance of a hernia after it has previously been repaired.

1. Does peer support before hernia surgery improve clinical outcomes?
2. Does an opt in pre-operative group/clinic information session improve Patient Reported Outcome Measures (PROMS) following hernia surgery?
